# Identification of new nucleotide sequences of the Glu-B1-1 gene encoding x-type glutenins in bread wheat

**DOI:** 10.18699/VJGB-23-52

**Published:** 2023-09

**Authors:** A.A. Galimova, B.R. Kuluev

**Affiliations:** Institute of Biochemistry and Genetics – Subdivision of the Ufa Federal Research Centre of the Russian Academy of Sciences, Ufa, Russia Federal Research Center the N.I. Vavilov All-Russian Institute of Plant Genetic Resources (VIR), St. Petersburg, Russia; Institute of Biochemistry and Genetics – Subdivision of the Ufa Federal Research Centre of the Russian Academy of Sciences, Ufa, Russia Federal Research Center the N.I. Vavilov All-Russian Institute of Plant Genetic Resources (VIR), St. Petersburg, Russia

**Keywords:** baking quality, high-molecular-weight glutenin subunits, Glu-1 genes, genotyping, хлебопекарные качества., высокомолекулярные субъединицы глютенина, гены Glu-1, генотипирование

## Abstract

Studies of the genetic base and polymorphism of bread wheat cultivars aimed at identifying alleles of genes associated with high baking and other economically valuable traits seem to be relevant, since bread wheat, along with all representatives of the Triticeae tribe, has a huge genetic potential for creating cultivars with high technological and rheological properties of grain flour. The aim of this study was sequencing and analysis of the nucleotide sequences of the Glu-B1-1 gene, and analysis of the predicted amino acid sequences of its protein product in three cultivars of bread wheat. Thus, in the course of genotyping cultivars and lines of bread wheat for the Glu-B1-1 gene, in the cultivars ‘Avesta’, ‘Leningradka krupnozernaya’ and line C-75094, previously undescribed changes in the size of amplifiable regions of the Glu-B1-1 gene for high-molecular weight glutenins were found. Comparative analysis of the nucleotide sequences of these genes with known sequences showed the presence of two deletions in ‘Avesta’ and C-75094 and the presence of seven single-nucleotide substitutions in ‘Leningradka krupnozernaya’. Alignment of the predicted Glu-B1 amino acid sequences of the studied accessions and the standard cultivar carrying the Glu-B1-a allele showed that deletions in the amino acid sequences of ‘Avesta’ and C-75094 accessions are localized in the central domain of the protein and affect the amount of tri-, hexa-, and nonapeptides, and in ‘Leningradka krupnozernaya’, a decrease in GQQ and PGQGQQ by one unit was revealed. In addition, substitutions of five amino acids were found in ‘Leningradka krupnozernaya’. Thus, we have found previously undescribed deletions and substitutions in the nucleotide sequences of the Glu-B1-1 gene for high-molecular-weight glutenins, which lead to changes in amino acid sequences in functionally important regions, namely, in the central domains of protein molecules. The identified mutations can be used for genotyping bread wheat cultivars.

## Abstract (ru)

Мягкая пшеница, наряду с другими представителями трибы Пшенициевых, обладает огромным генетическим потенциалом для создания сортов с высокими технологическими и реологическими свойствами муки. Поэтому исследования генетической базы полиморфизма сортов мягкой пшеницы и выявление аллелей генов, ассоциированных с высокими хлебопекарными признаками, представляются актуальными. Цель данной работы – анализ нуклеотидных последовательностей гена субъединиц x-типа высокомолекулярных глютенинов Glu-B1-1 и анализ предсказанных аминокислотных последовательностей его белкового продукта у трех генотипов мягкой пшеницы. В ходе генотипирования по гену Glu-B1-1 у сортов мягкой пшеницы Авеста, Ленинградка крупнозерная и линии С-75094 были обнаружены ранее не описанные изменения в размерах амплифицируемых участков. Сравнительный анализ нуклеотидных последовательностей этих генов с известными опубликованными последовательностями показал наличие двух делеций у генотипов Авеста и С-75094 и семи однонуклеотидных замен у сорта Ленинградка крупнозерная. Выравнивание предсказанных аминокислотных последовательностей Glu-В1 рассматриваемых генотипов и стандартного сорта, несущего аллель Glu-B1-а, показало, что делеции в аминокислотных последовательностях у сорта Авеста и линии С-75094 локализуются в центральном домене белка и влияют на количество три-, гекса- и нонапептидов. У сорта Ленинградка крупнозерная было выявлено уменьшение количества трипептида GQQ и гекса- пептида PGQGQQ на одну единицу; кроме того, выявлены замены пяти аминокислот. Таким образом, нами обнаружены ранее не описанные делеции и замены в нуклеотидных последовательностях гена высокомолекулярных глютенинов Glu-B1-1, которые приводят к изменениям аминокислотных последовательностей в функционально значимых участках, а именно в центральных доменах белковых молекул. Выявленные мутации могут быть использованы при генотипировании сортообразцов мягкой пшеницы.

## Introduction

High-molecular-weight glutenin subunits (HMW-GS) play an important role in determining the viscoelastic properties of bread wheat grains as they contribute to the formation of larger gluten polymers and are major determinants of dough elasticity (Shewry et al., 1989, 1992, 1995, 1997). Therefore, the characterization of the HMW-GS composition is an important task in bread wheat breeding programs aimed at improving grain quality. This makes it possible to predict the baking qualities of bread wheat cultivars (Payne, 1987; Nucia et al., 2019)

Until recently, SDS electrophoresis of storage proteins was the main method for determining HMW-GS composition, which revealed a huge allelic diversity of HMW-GS in the Triticeae tribe. For example, to date, 52 alleles have been identified for the Glu-A1 locus of subgenome A, 83 alleles for the Glu-B1 locus of subgenome B, and 70 alleles for the Glu-D1 locus of subgenome D (McIntosh et al., 2013)

Recently, protein SDS electrophoresis has been replaced by methods of molecular genetics, which make it possible to distinguish subunits of high-molecular-weight glutenins with similar molecular weights at the genetic level (Vafin et al., 2018; Nucia et al., 2019). However, the nucleotide sequences of most HMW-GS alleles identified using protein electrophoregrams have not been characterized and deposited in databases still. Studies aimed at determining the nucleotide sequences of alleles of genes associated with high or low grain quality are relevant, since their results can be used in markerassisted and genomic selection of bread wheat

In the course of genotyping 95 bread wheat cultivars according to the composition of storage protein genes (Galimova et al., 2023), we identified genotypes carrying previously unknown x-type nucleotide sequences encoded by the Glu- B1-1 locus (designation in accordance with the Catalogue of Gene Symbols for Wheat (McIntosh et al., 2013)). They were found in cultivars Avesta, Leningradka krupnozernaya and line C-75094. This study describes these new deletions and nucleotide substitutions, as well as some characteristics of the amino acid sequences’ predicted fragments of the subun

## Materials and methods

The materials of the study were winter bread wheat cultivars Sterlinskaya (used as a control sample – a cultivar carrying the allele of the x-type subunit – Bx7), Avesta, spring cultivar Leningradka krupnozernaya and line C-75094, obtained from the VIR collection. According to the VIR, the cultivar Leningradka krupnozernaya and the line C-75094 have low baking qualities. In accordance with the data given in the Russian State Register of Breeding Achievements (https://reestr. gossortrf.ru/sorts/9358556/, accessed 10/15/2022), the Avesta cultivar is characterized by good baking qualities

Isolation of total DNA from dried bread wheat leaves was performed using CTAB (Doyle J.J., Doyle J.L., 1987). The BxF/BxR primer pair was used to amplify the Glu-B1-1 gene fragment (Ma et al., 2003). The BxF forward primer is annealed at two regions of the Glu-B1-1 gene, forming during PCR, together with the reverse primer, two reaction products with sizes of 766 and 630 bp. DNA amplification was carried out according to the program: initial denaturation for 5 min at 95 °C; 35 cycles of denaturation at 95 °C for 40 sec, primer annealing at 58 °C for 40 sec, elongation at 72 °C for 1 min and final elongation for 3 min at 72 °C. Amplification results were visualized in 1.6 % agarose gels with DNA fragment length markers of 100 bp (Evrogen, Russia).

For sequencing PCR products, an average of 500 ng of each PCR product obtained above was used. The products were purified using the following reaction: 1 U alkaline phosphatase (NEB, USA) and 10 U exonuclease I (NEB, USA) in a final volume of 10 μl at 37 °C for 15 min, followed by enzyme inactivation at 85 °C for 15 min. 1 μl (~50 ng) of each purified sample was directly used as a template for sequencing. The reaction was set up using 10 pmol primer and 0.5 μl BigDye™ Terminator v3.1 Ready Reaction Mix in a final volume of 10 μl. The cycles of the sequencing reaction: denaturation at 96 °C for 10 sec, primer annealing at 58 °C for 5 sec, elongation at 60 °C for 4 min for all 30 cycles. Fluorescently labeled PCR products were analyzed using an Applied Biosystems 3500 genetic analyzer (Thermo Fisher Scientific, USA).

Three biological replicates were used when sequencing fragments of the studied genes of each sample. Sequencing was performed at both ends using primers BxF and BxR. Further, for each sample, by aligning the three obtained sequences, one consensus sequence was compiled. This procedure was carried out primarily to avoid possible errors in sequencing. Alignment of nucleotide sequences by the Clustal W method and detection of putative mutations were performed using the MEGA program version 11.0.8 (Molecular Evolutionary Genetics Analysis version 11).

## Results

**Fig. 1.**
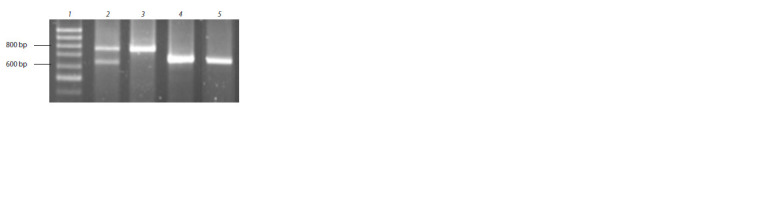
Electrophoregram of the Glu-B1-1 gene fragment amplification results with primers BxF/BxR: 1 – 100 bp DNA ladder (Eurogen, Russia); 2 – cultivar Sterlinskaya with expected amplicon sizes of 766 + 630 bp; 3 – cultivar Leningradka krupnozernaya (766 bp); 4 – cultivar Avesta (~669 bp); 5 – line C-75094 (~669 bp).

In the course of genotyping 95 cultivars and lines of bread wheat for the Glu-1 genes of subgenomes A, B, and D with genome-specific primers (Galimova et al., 2023), we found previously undescribed deletions in the nucleotide sequence of the Glu-B1 gene encoding HMW-GS x-type (Glu-B1-1). When analyzing the allelic state of the Glu-B1-1 gene, the expected products of the amplification reaction were 766 and 630 bp amplicons. The production of reaction products of the indicated sizes would show that the cultivar carries the allele of the x-type subunit Bx7 (in this study, the Sterlinskaya cultivar was taken as a control). However, in the case of three samples (Avesta, Leningradka krupnozernaya cultivars and C-75094 line), during genotyping, reaction products were found that differed from those expected – one reaction product instead of two with a size of 766 and ~669 bp, not previously described in the literature. Amplification of the genomic DNA of the Leningradka krupnozernaya cultivar produced only one reaction product 766 bp in size. Amplification of the DNA of the Avesta cultivar and the C-75094 line also resulted in the formation of one reaction product, while an amplicon larger than 630 bp and less than 700 bp was detected on the electrophoregram. There are data in the literature on the formation of PCR products with a size of 669 bp when using the BxF/BxR primer pair (Ma et al., 2003). This gave reason to believe that the PCR products of the Avesta and C-75094 genotypes represent the previously described fragment of the Glu-B1-1 gene with a size of 669 bp (Galimova et al., 2023). Therefore, below, the size of this PCR product was designated as ~669 bp (Fig. 1, Table 1).

**Table 1.**
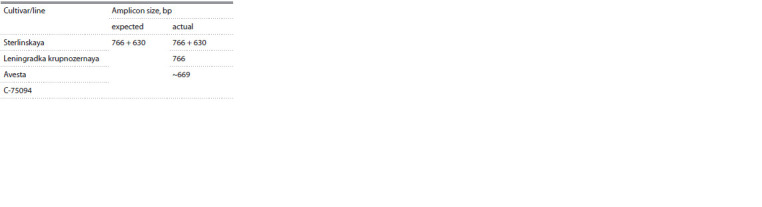
Expected and actual amplicon lengths during genotyping of the studied bread wheat genotypes using BxF/BxR primers

**Fig. 2.**
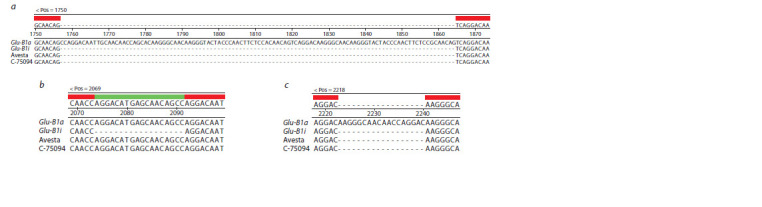
Alignment of the nucleotide sequences of the Glu-B1-1 gene fragments of the Avesta and C-75094 genotypes with the Glu-B1-1 nucleotide sequences of the alleles Glu-B1a (GenBank BK006773) and Glu-B1i (GenBank AB263219). a – deletion (1757–1864 nucleotides); b – deletion (2074–2091 nucleotides, this deletion distinguishes the nucleotide sequences of Glu-B1-1 of the Glu-B1i allele and the studied genotypes Avesta and C-75094); c – deletion (2223–2240 nucleotides).

To determine the nucleotide sequences of the Glu-B1-1 gene detected fragments, their sequencing was carried out. Comparative analysis of the nucleotide sequences of the Glu-B1-1 gene fragments of the Avesta and C-75094 genotypes with known sequences from the GenBank database containing annotated DNA and RNA sequences did not reveal complete identity between them. Alignment of the nucleotide sequence of the Glu-B1-1 gene fragments of the studied samples (C-75094, Avesta) showed their similarity to the x-type subunit of the i allele, which has three deletions relative to the a allele. In the samples we studied, only two of them were identified (Fig. 2, a–c). Thus, the size of the amplified and sequenced fragment of the Glu-B1-1 gene was 687 bp.

**Fig. 3.**
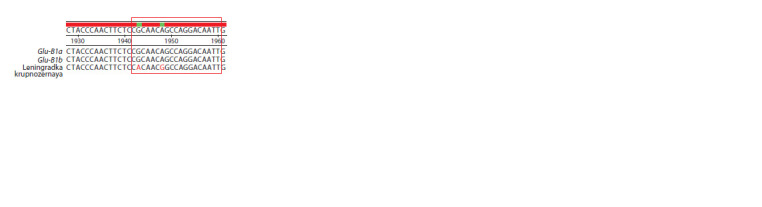
Single nucleotide substitutions (G→A and A→G) in the nucleotide sequence of the Glu-B1-1 gene of the Leningradka krupnozernaya cultivar at the site of the BxF forward primer annealing. The annealing site of the BxF forward primer is marked with a red frame.

Analysis of the nucleotide sequence of the Glu-B1-1 gene fragment of the cultivar Leningradka krupnozernaya, for which an amplification product of 766 bp was detected, showed that two single-nucleotide substitutions occurred in one of the two annealing regions of the BxF forward primer (G→A and A→G), which probably prevent annealing of the forward primer (Fig. 3). Presumably, as a result of this, only one reaction product is formed, instead of the expected two.

Since the Glu-B1-1 gene of the studied genotypes (Avesta, Leningradka krupnozernaya, С-75094) was not completely sequenced, it was supplemented at both ends with flanking regions of the Glu-B1a allele (GenBank BK006773) for comparative analysis of their amino acid sequences. Thus, the analysis of the predicted amino acid sequences of the Glu-B1-1 protein of the studied genotypes will be carried out on the basis of data obtained as a result of sequencing of the fragment of the Glu-B1-1 gene.

The central part of the Glu-B1-1 protein is represented by repeating motifs of the tri-, hexa-, and nonapeptides GQQ, PGQGQQ, and GYYPTSPQQ. Despite the fact that the amino acid sequences of the studied genotypes Avesta and C-75094 have a number of amino acids different from the number of amino acids of the Glu-B1i allele carrier cultivar, all three compared amino acid sequences have the same number of tri-, hexa-, and nonapeptides repeats (Table 2).

**Table 2.**
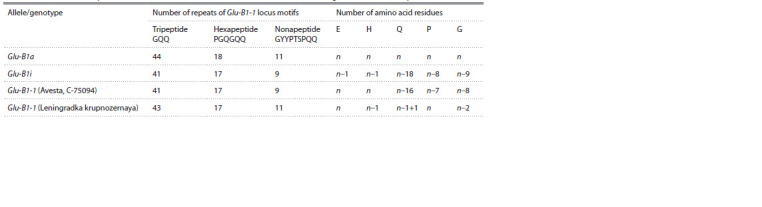
The number of repeated HMW-GS motifs and amino acid residues in the studied region of the Glu-B1-1 protein Note. Amino acid residues abbreviations: E – glutamic acid, H – histidine, Q – glutamine, P – proline, G – glycine. n – number of amino acid residues of the Glu-B1-1 protein of the genotype (cultivar) carrying the Glu-B1a allele; n–1+1 – substitution leading to the formation of the amino acid glutamine (Н→Q), and substitution of the amino acid glutamine with another amino acid (Q→R).

**Fig. 4.**
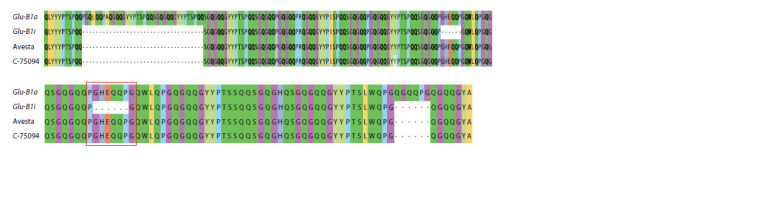
The results of alignment of the predicted amino acid sequences of the Glu-B1-1 protein of cultivars carrying the alleles Glu-B1a, Glu-B1i, and the studied samples Avesta, C-75094. The red frame highlights the region where the amino acid sequences of the studied Avesta and C-75094 samples differ from the amino acid sequence of the protein encoded by the Glu-B1i allele.

Significant differences in the number of amino acid residues and motifs are observed when comparing the amino acid sequences of the three mentioned cultivars and lines with the amino acid sequence of the cultivar carrying the Glu-B1a allele. Thus, in the amino acid sequence of the Glu-B1a allele, there are 44 GQQ tripeptides, 18 PGQGQQ hexapeptides, and 11 GYYPTSPQQ nonapeptides, while in the amino acid sequences of the cultivar carrying the Glu-B1i allele and in the studied samples Avesta and C-75094, the number of peptides decreases by 3, 1 and 2 motives, respectively (Fig. 4, see Table 2)

Analysis of the number of amino acids in the predicted amino acid sequences of the four compared genotypes showed a decrease in the number of E (glutamic acid), H (histidine), Q (glutamine), P (proline), G (glycine) residues in the studied samples of Avesta, C-75094 and in the cultivar with the Glu- B1i allele, compared with the cultivar carrying the Glu-B1a allele (see Table 2). Thus, a significant difference in the number of glutamines was found in the amino acid sequences of the Glu-B1a allele and the amino acid sequences of the other studied samples: n–18 glutamine residues in the Glu-B1i allele and n–16 in the amino acid sequences of the Avesta and C-75094 genotypes. In addition, the Glu-B1-1 amino acid sequences of the Avesta and C-75094 samples show differences in the numbers of prolines (n–7) and glycines (n–8). The cultivar carrying the Glu-B1i allele (GenBank AB263219), in addition to glutamine, differs in the number of 4 more amino acids: glutamic acid, histidine, proline, and glycine (see Table 2, Fig. 4). From the analysis of the amino acid sequence predicted fragments, it can be seen that the sequenced glutenin fragment of the studied samples (Avesta, Leningradka krupnozernaya, C-75094) lacks newly formed cysteine residues that are significant for the formation of disulfide bonds (see Table 2, Fig. 4).

## Discussion

The HMW-GS gene polymorphism is most likely one of the reasons for the high genetic variability of bread wheat traits that affect the technological and rheological properties of flour, and, as a result, baking quality (Patil et al., 2015; Ravel et al., 2020). In the course of genotyping various cultivars and lines of bread wheat at the Glu-B1-1 locus, we identified amplicons, the nucleotide sequence lengths of which did not correspond to the expected ones. The changes in the nucleotide composition of the Glu-B1 x-type subunit gene found in this study have not been previously described.

HMW-GS consist of N- and C-terminal domains and a central domain that consists of repeating motifs (Shewry et al., 1992). The N- and C-terminal domains contain more charged residues than the central domain and include most or all of the cysteine residues present in the subunits. Repeat domains are characterized by tri-, hexa-, and nonapeptide motifs in x-type subunits (GQQ, PGQGQQ and GYYPTSPQQ) and hexa- and nonapeptide repeats in y-type subunits (PGQGQQ and GYYPTSLQQ) (Tatham et al., 1990). Thus, two features of the HMW-GS structure, the number and distribution of disulfide bonds, as well as the properties and interactions of the repeating motifs of the central domain, can be related to the determination of protein elasticity (Kohler et al., 1994).

Disulfide bonds are extremely important for the structure of gluten and are significant factors in determining the viscoelastic and rheological properties of the dough (Lindsay et al., 2000; Li et al., 2016). Intra- and intermolecular disulfide bonds form between cysteine residues (Wang et al., 2021). For the predicted fragments of the amino acid sequences of the studied genotypes, an analysis of the cysteine residues content was carried out, which showed no changes in their number.

Although interchain disulfide bonds are critical for stabilizing HMW-GS polymers, nuclear magnetic resonance studies indicate that hydrogen bonds mediated by glutamine side chains may also play an important role in gluten structure stabilization (Belton, 1994; Belton et al., 1995). A high content of glutamine residues has a high ability to form both intra- and intermolecular hydrogen bonds and positively affect dough elasticity (Belton, 1999; Guo et al., 2019). In the genotypes studied, changes in the content of glutamine were found (see Table 2). Note that samples Avesta and C-75094 are characterized by the presence of a greater number of glutamine residues compared to the cultivar carrying the Glu-B1i allele.

Variations in the central repeat domain of glutenin proteins are the main reasons for differences in the size of its subunits (Anderson, Greene, 1989; Halford et al., 1992; Shewry et al., 1992; D’Ovidio et al., 1995), which was also shown in our study. It can be seen from the analysis of the central region of the predicted HMW-GS protein in samples Avesta and C-75094 that they differ from the known amino acid sequence of cultivars carrying the Glu-B1a allele in the number of motifs (all three types), and, accordingly, in the number of amino acids. They differ from the amino acid sequence of the Glu-B1i allele only in the number of amino acids. Thus, the number of central domain motifs in samples Avesta and C-75094 and in the Glu-B1i allele is the same and equals 41 tri-, 17 hexa-, and 9 nonapeptides, but the number of amino acid residues in them is different (see Table 2, Fig. 4). For the spring cultivar Leningradka krupnozernaya, a decrease in the amount of tri- and hexapeptides was shown compared to the amino acid sequence of the cultivar carrying the Glu-B1a allele (see Table 2). Thus, the amino acid sequences of the cultivars Avesta, Leningradka krupnozernaya and line C-75094 have a lower number of motif repeats compared to the cultivar carrying the Glu-B1а allele.

It is known that the length of the central domain, that is, the number of repetitions of its motifs, affects dough elasticity (Gianibelli et al., 2001). It is possible that in the genotypes Avesta, C-75094, and Leningradka krupnozernaya, one of the factors of low baking qualities is a decrease in the number of repeats of the GQQ, PGQGQQ, GYYPTSPQQ motifs and the number of amino acid residues of glutamine and glycine in the Glu-B1-1 protein.

## Conclusion

The study describes previously unknown nucleotide sequences of the x-type HMW-GS gene, Glu-B1-1, which were found during the genotyping of Glu-1 gene alleles in the Avesta, Leningradka krupnozernaya cultivars and the С-75094 line. The identified mutations can be used for genotyping cultivars and lines of bread wheat for the HMW-GS genes. They can also be proposed as DNA markers in breeding, but this requires further detailed studies on the effect of the identified mutations on the baking quality of grain. Differences in the nucleotide sequences of the Glu-B1-1 gene lead to changes in the predicted amino acid sequences of their proteins. Changes in the number of tri-, hexa-, and nonapeptide repeats of the central domain of the protein were predicted in the studied genotypes, and changes in the number of glutamine and glycine were revealed. Since the length of the central domain, as well as the amino acid composition of repetitive motifs, are significant in determining the intra- and intermolecular interactions of a protein molecule, the results of the study can be taken into account when analyzing the viscoelastic properties of the dough and economically valuable traits in the studied cultivars and lines.

## Conflict of interest

The authors declare no conflict of interest.
